# Decolorization of RhB dye by manganese oxides: effect of crystal type and solution pH

**DOI:** 10.1186/s12932-015-0024-2

**Published:** 2015-07-25

**Authors:** Hao-Jie Cui, Hai-Zheng Huang, Baoling Yuan, Ming-Lai Fu

**Affiliations:** Institute of Urban Environment, Chinese Academy of Sciences, Xiamen, 361021 China; College of Civil Engineering, Fuzhou University, Fuzhou, 350116 China; College of Civil Engineering, Huaqiao University, Xiamen, 361021 China

**Keywords:** RhB, Manganese oxides, Crystal structure, Solution pH, Oxidation

## Abstract

**Background:**

Organic dye pollution in water has become a major source of environmental pollution. Mn(III/IV) oxides have attracted a great deal of attention to remove organic dye pollutants due to their unique structures and physicochemical properties. Numerous studies have reported the removal of dye by various Mn(III/IV) oxides through catalytic degradation and adsorption. The crystalline structures of manganese oxides and solution pH may exert substantial impact on the removal of dyes. However, few studies have focused on the oxidative degradation of RhB dye using Mn(III/IV) oxides with different crystal structures during a spontaneous reaction. In the present study, three manganese oxides with different crystal type (α-MnO_2_, β-MnO_2_, and δ-MnO_2_) were prepared by refluxing process to decolorize RhB dye in various pH solutions.

**Results:**

The results showed that the decolorization efficiencies of RhB for the three manganese oxides all increase with decrease solution pH. α-MnO_2_ exhibited highest activity and could efficiently degrade RhB at pH 2–6. The degradation of RhB by β-MnO_2_ and δ-MnO_2_ could be observed at pH 2–3, and only little adsorption RhB on manganese oxides could be found at pH 4–6. The UPLC/MS analysis suggests that the decolorization of RhB by manganese oxides consists of three main stages: (1) cleavage of the ethyl groups from RhB molecular to form Rh; (2) further destruction of –COOH and –CNH_2_ from Rh to form the small molecular substances; (3) mineralization of the small molecular substances into CO_2_, H_2_O, NO_3_^−^ and NH_4_^+^.

**Conclusions:**

Overall, these results indicate that α-MnO_2_ may be envisaged as efficient oxidants for the treatment of organic dye-containing wastewater under acid conditions.

**Electronic supplementary material:**

The online version of this article (doi:10.1186/s12932-015-0024-2) contains supplementary material, which is available to authorized users.

## Background

**Scheme 1:**
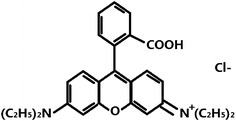
Molecular structure of RhB.

Nowadays, organic dye pollution in water has become a major source of environmental pollution due to the fast development of dye industry [1, 2]. Discharging of the residual dyes creates acute problems to the ecosystem and human health [3]. For example, Rhodamine B (RhB), as an important cationic xanthene dye (Scheme 1), has been extensively used in textile, printing, and photographic industries. It has been found to possess carcinogenicity, neurotoxicity, and chronic toxicity towards humans and animals [4]. Therefore, it is necessary to destroy the dyes from industrial effluents before they become detrimental to the natural environments.

In the recent years, Mn(III/IV) oxides have attracted a great deal of attention to remove organic dye pollutants due to their unique structures and physicochemical properties [5–7]. The removal efficiencies of the dye pollutants by Mn(III/IV) oxides are dependent upon their crystallographic forms, which could display layered or tunnel structures through different arrangements of MnO_6_ octahedra [8, 9]. Up to now, numerous studies have reported the removal of dye by various Mn(III/IV) oxides through catalytic degradation and adsorption [5, 10–12]. Few studies have focused on the oxidative degradation of RhB using Mn(III/IV) oxides with different crystal structures during a spontaneous reaction. Moreover, as reported in the previous literatures, the Mn(III/IV) oxides were commonly prepared by different methods including refluxing process, hydrothermal method, and calcination method [12–14]. The Mn(III/IV) oxides prepared using different methods often display different reactivity.

In the present work, three Mn(III/IV) manganese oxides (α-MnO_2_, β-MnO_2_, and δ-MnO_2_) with different crystal structures were prepared by similar refluxing process, and the products were used to decolorize RhB in various pH solutions to investigate the effects of crystal type of manganese oxides and solution pH on the removal efficiencies and mechanisms of RhB dye from waters.

## Results and discussion

### Characterizations of the synthetic manganese oxides

**Fig. 1:**
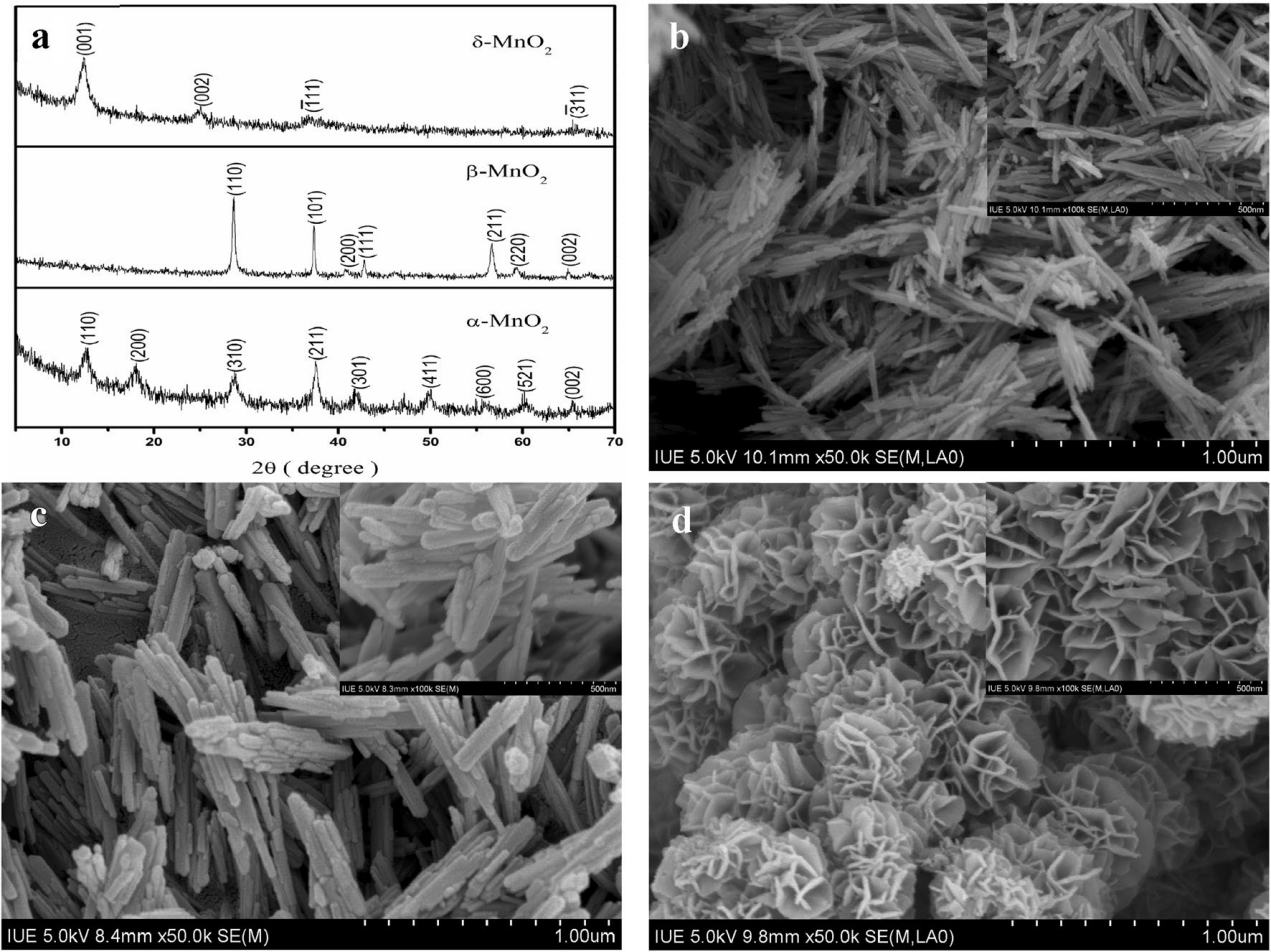
XRD patterns (**a**) and SEM images of the synthetic manganese oxides: **b** α-MnO_2_; **c** β-MnO_2_; **d** δ-MnO_2_.

Figure 1a shows the XRD patterns of the as-prepared manganese oxides. All the diffraction peaks could be readily indexed to α-MnO_2_ (JCPDS 44-1386), β-MnO_2_ (JCPDS 24-0735) and δ-MnO_2_ (JCPDS 80-1098), respectively. The results indicate that purely crystalline α-MnO_2_, β-MnO_2_ and δ-MnO_2_ were successfully synthesized. The SEM images show that the as-prepared α-MnO_2_ samples consist of needle-like nanostructures with a diameter of 10–30 nm and a length of 300–1,000 nm (Fig. 1b). The synthetic β-MnO_2_ samples exhibit rod-like shape with a diameter of 50–100 nm and a length of 200–500 nm (Fig. 1c). The δ-MnO_2_ shows a three dimensional hierarchical microsphere with a diameter ranging from 300 to 500 nm, and the microspheres consist of nanoplates with a thickness of an approximately 10 nm (Fig. 1d). The BET surface area, average Mn oxidation state (AOS), and the point of zero charge (PZC) of the three manganese oxides were also characterized and the results were listed in Table 1.

**Table 1:** Selected properties of the synthetic manganese oxides

Samples	BET surface area (m^2^/g)	Mn AOS	PZC
α-MnO_2_	83.5	3.89	4.7
β-MnO_2_	27.9	3.96	3.8
δ-MnO_2_	40.1	3.71	3.4

### Decolorization of RhB dye by the manganese oxides at various pH values

**Fig. 2:**
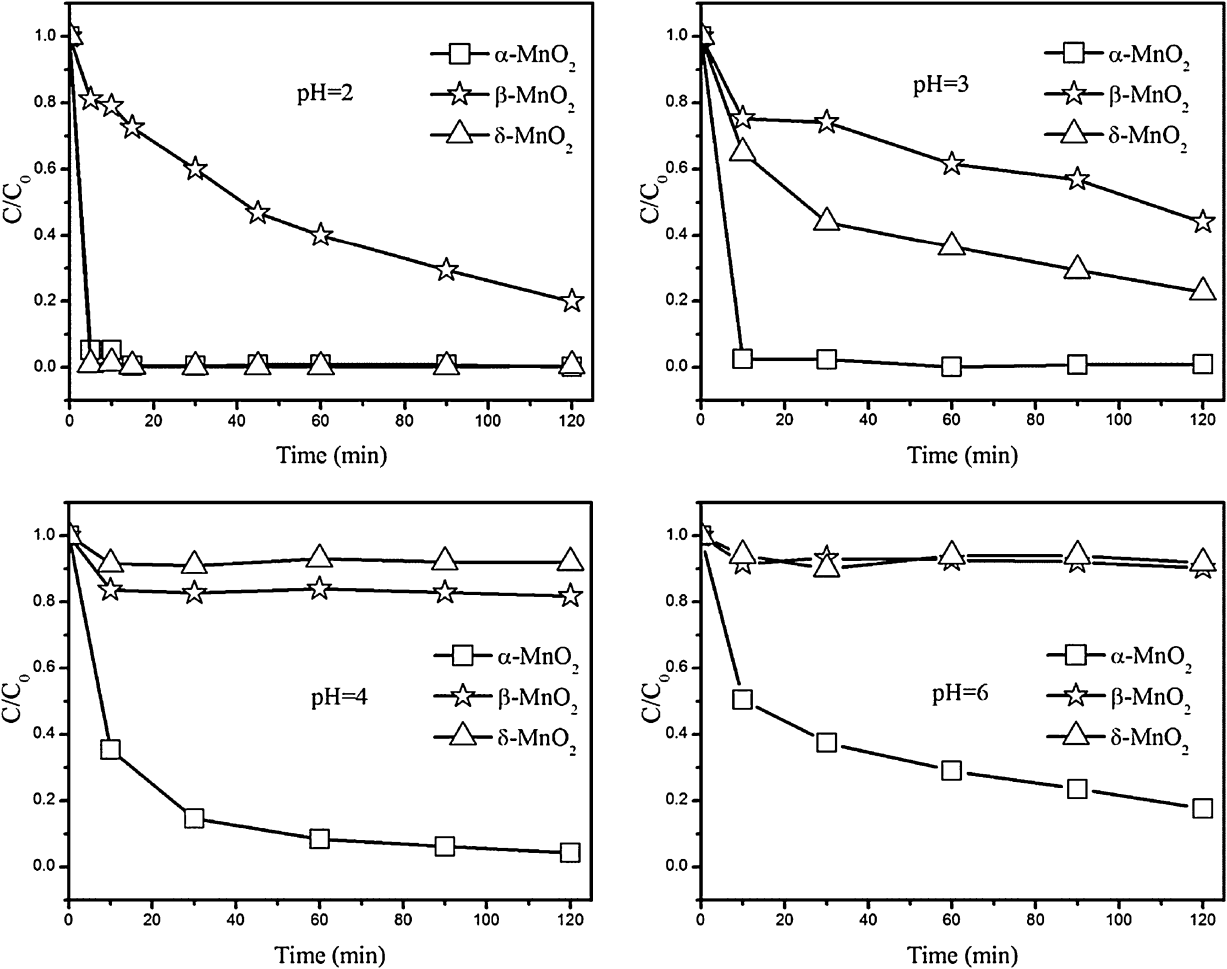
The effect of pH on the decolorization of RhB by the three manganese oxides.

The results for decolorization of RhB dye by the three manganese oxides at different pH were shown in Fig. 2. In detail, at pH 2, α-MnO_2_ and δ-MnO_2_ both possessed high efficiency to decolorize RhB, and the degree of decolorization reached above 90% within 5 min. However, the decolorization of RhB by β-MnO_2_ is far slower than that of α-MnO_2_ and δ-MnO_2_, and only about 80% of initial of RhB was decolorized within 120 min. At pH 3, more than 90% of the RhB could be decolorized by α-MnO_2_ within 10 min, which is much faster than that of δ-MnO_2_ and β-MnO_2_. After reaction of 120 min, the decolorization efficiencies of RhB by δ-MnO_2_ and β-MnO_2_ are only about 80 and 50%, respectively. During the reaction at pH 4, only less than 20% of RhB could be decolorized by δ-MnO_2_ and β-MnO_2_ within 120 min, and the decolorization proceeds with high efficiency is still observed for α-MnO_2_. Further increasing pH to 6, no obvious dye decolorization is observed for δ-MnO_2_ and β-MnO_2_ after 120 min, the degree of decolorization for α-MnO_2_ decreased to about 80%. These results indicated that the decolorization efficiency of RhB by manganese oxides depend on the crystal type and solution pH, and α-MnO_2_ presents highest capability to decolorize RhB in pH 2-6.

UV–vis absorption spectra of the RhB solution treated with the three manganese oxides are shown in Additional file 1: Figure S2. For α-MnO_2_, the intensity of the RhB peaks decreased sharply within 5–10 min, and the peak of 554 nm obviously shifted to 500 nm, which indicates that the decolorization of RhB by α-MnO_2_ is mainly attributed to the decomposition reaction at pH 2–6 [15, 16]. Meanwhile, the same blue shift of 554 nm peak was observed at low pHs for δ-MnO_2_ (pH 2 and 3) and β-MnO_2_ (pH 2). However, only a weak decrease of the intensity of the RhB peaks, and no peak shifts were found at pH 4 and pH 6. These results implied that the decomposition reaction only occurs at pH ≤ 3 and adsorption is responsible to the decolorization of the RhB at pH > 3. Therefore, the decolorization mechanism of RhB by manganese oxides depends on the crystal type of the oxides and the solution pH.

In this investigation, the three crystallographic MnO_2_ showed different activities for RhB degradation, which can be related to the variation in crystalline structure, AOS, BET surface area and other physicochemical properties. At low pHs, the crystal stability of the layered structure of δ-MnO_2_ is weaker than that of the tunnel structures of α-MnO_2_ and β-MnO_2_, which is beneficial to the redox reaction between Mn(VI/III) and RhB. This may account for its faster oxidization of RhB at pH 2 than α-MnO_2_ and β-MnO_2_. With increasing solution pH to 4, the adsorption became fully responsive to the decolorization of RhB for δ-MnO_2_. However, the oxidation of RhB still could be found for α-MnO_2_ and β-MnO_2_ at pH 4. This may result in their higher decolorization rates. Compared with tunnel structure of α-MnO_2_ and β-MnO_2_, two-tunnel structured α-MnO_2_ showed higher activity than the single-tunnel structured β-MnO_2_ due to the more exposure of MnO_6_ edges [17], although the β-MnO_2_ has a higher AOS (3.96). Moreover, the BET surface area of β-MnO_2_ is much smaller than that of α-MnO_2_ and δ-MnO_2_, which means higher crystallization degree for β-MnO_2_. Thus, the activation energy to break the crystal structure is more than that of α-MnO_2_ and δ-MnO_2_ during redox reactions. This may explain the lower oxidation capability of β-MnO_2_ for RhB at low pH solution.

The variation of the solution pH influenced the surface charge properties of the dye and the manganese oxides as well as their interactions. As shown in Scheme 1, the molecular of RhB contains carboxylic group and amino group. The carboxylic group exists in the protonated state by decreasing the solution pH beyond the pK_S2_ value, which corresponds to 3.22, and the amino group is protonated only under very weakly basic conditions with the pK_B_ value being 13.75 [18]. At pH ≤ 3.22, the positively charged lead manganese oxides surface interacts more attractively with the uncharged carboxylic acid than with the positively charged amino group. At pH > PZCs of manganese oxides, the attractive interaction is established between the positively amino group and the negatively charged surface of manganese oxides. In our experiments, the decolorization efficiencies of RhB for the three manganese oxides all increased with decrease solution pH, which could be attributed to the increase of the redox potential, electron transfer, and the surface charge density of MnO_2_ at lower pH values. Thus, the oxidation rate of RhB by the manganese oxides is dependent upon their physicochemical parameters, crystal structures, and solution pHs.

### Reduction and dissolution of the synthetic manganese oxides by RhB dye

**Fig. 3:**
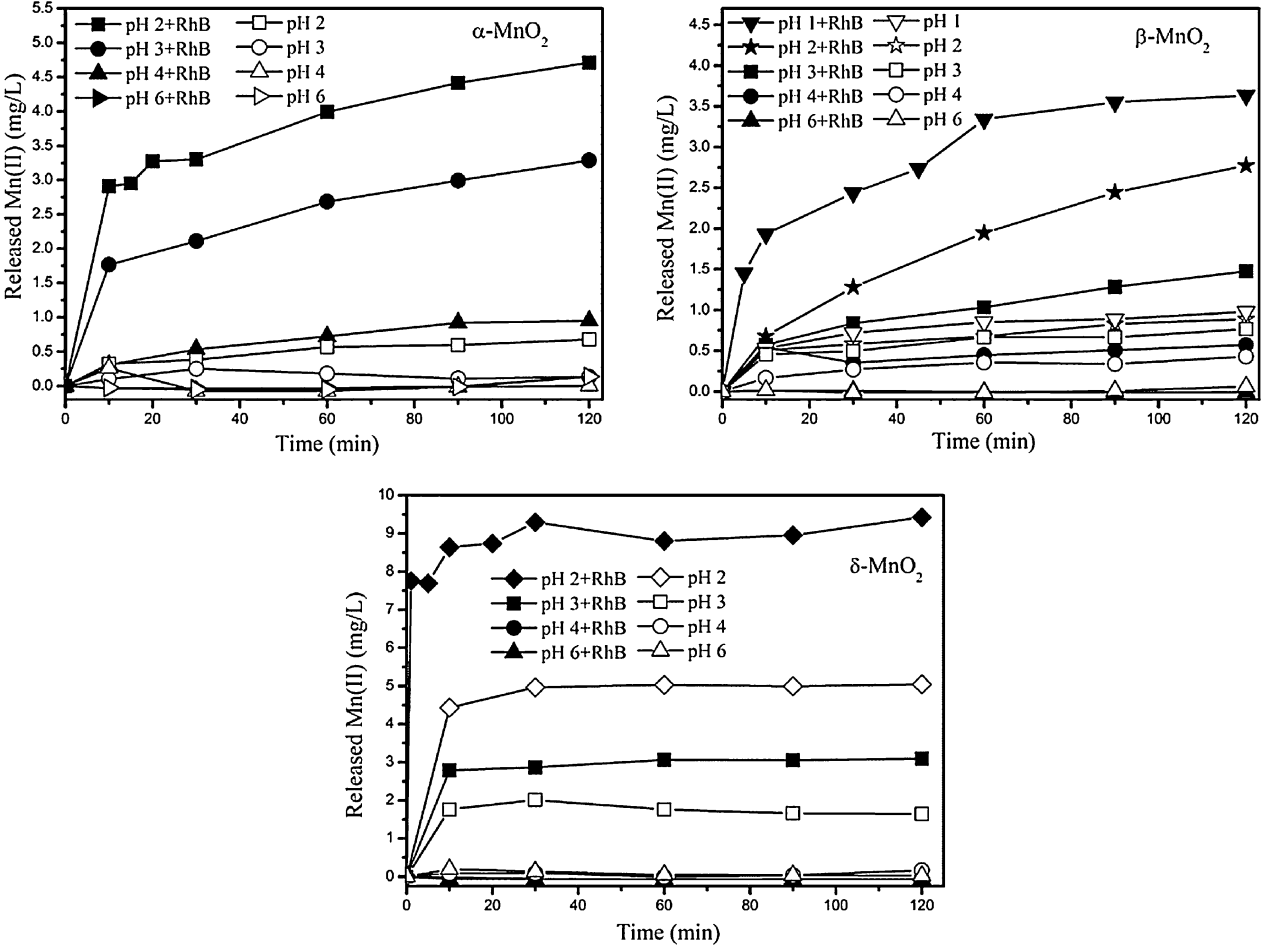
Dissolution behavior of the manganese oxides with and without RhB at different pH.

The degradation of organic compounds by manganese oxides commonly accompany release of Mn(II) from reductive dissolution of manganese oxides [19, 20]. Figure 3 shows the dissolution behavior of the three manganese oxides with and without RhB at different pH. When pH > PZC (MnO_2_), only a small amount of released Mn(II) could been determined in the solutions, and the amount of Mn(II) with RhB treatments are closed to that of treatments without RhB, indicating that Mn(II) is mainly derived from the manganese oxides through slightly dissolving or ion-exchanging by H^+^. When pH < PZC (MnO_2_), the amount of released Mn(II) increased obviously with decreasing pH, and the amount of released Mn(II) with RhB treatments are much higher than that of the treatments without RhB, suggesting that the redox reaction occurs in the case of pH < PZC (MnO_2_). Moreover, it is observed that the amount of the released Mn(II) from δ-MnO_2_ was much higher than that of α-MnO_2_ and β-MnO_2_ at pH 2, which could attribute to the low AOS of Mn and the layered structures of δ-MnO_2_ [7]. To investigate the components and structures of the residual manganese oxides, the XRD analysis was performed. The XRD patterns of the manganese oxides before and after duplicating the degradation reaction five times show no obvious differences, indicating that the unchanged structure of the manganese oxides after the surface oxidative degradation. These results demonstrate that the degradation of RhB dye dose not generate a new manganese oxide, and the Mn(III/IV) is ultimately reduced to Mn(II) which is then released into the solution.

### Degradation mechanism of RhB by manganese oxides

To explore the mechanisms of the pH and crystal type on the RhB removal using manganese oxides, the intermediates of the reaction were analyzed using UPLC/MS technique. It is found that five peaks were observed at 3.05, 4.21, 4.70, 5.68, and 6.99 min in the UPLC/MS spectrum, which respectively correspond to the m/z = 331, 359, 387, 443, and 258. The signal at m/z = 443 could assign to the RhB parent ion [14]. The peaks at m/z = 387, 359, and 331 could refer to the formation of *N*-ehyl-*N*′-ethyl-rhodamine 110 (MMRh), *N*-ethyl-rhodamine 110 (MRh) and rhodamine 110 (Rh) intermediates, which predicted by the cleavage of two, three, and four ethyl group from RhB molecule, respectively [21–24]. The appearance of a peak at m/z = 258 could be due to cleavage of –COOH and –CNH_2_ from Rh (m/z = 331). Additionally, the TOC concentration of the solution decreased from 18.2 mg/L of the initial solution to 2.3 mg/L of treatment for α-MnO_2_ at pH 2. Moreover, NO_3_^−^ and NH_4_^+^ could be detected in the treated solutions and the concentrations of them are 0.86 and 0.23 mg/L, respectively. These results indicated that most of the RhB could be degraded completely by manganese oxides. Based on the above experimental observations, a plausible degradation mechanism of RhB was proposed (Additional file 1: Figure S6). It is suggested that the decolorization of RhB by manganese oxides consists of three main stages: (1) cleavage of the ethyl groups from RhB molecular to form Rh; (2) further destruction of –COOH and –CNH_2_ from Rh to form the small molecular substances; (3) mineralization of the small molecular substances into CO_2_, H_2_O, NO_3_^−^ and NH_4_^+^.

**Fig. 4:**
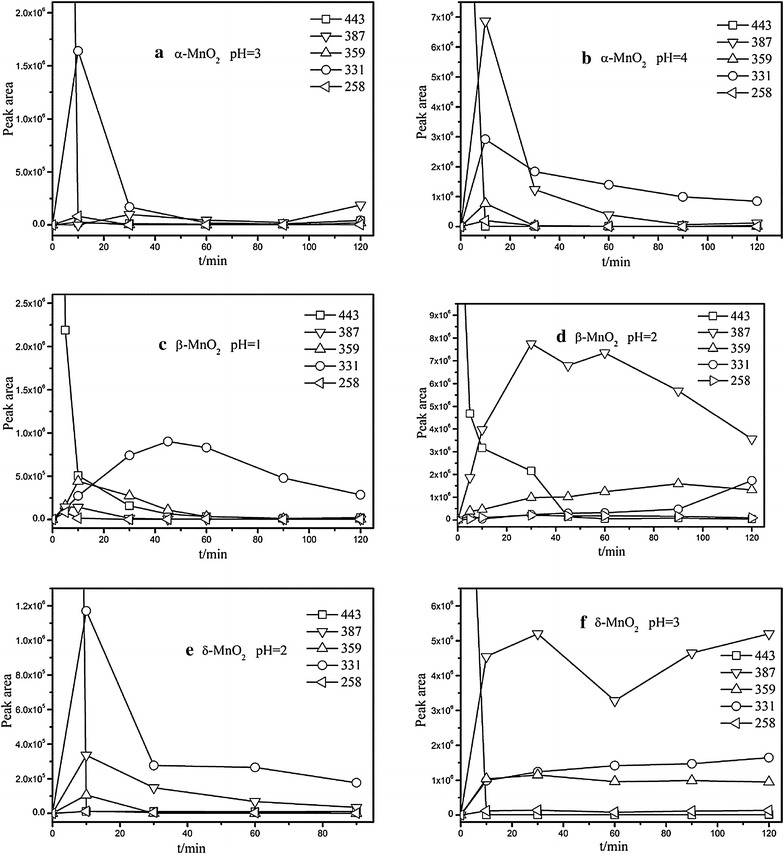
Measured intensities of RhB and the intermediates by UPLC/MS in function of the reaction time for the three manganese oxides at different pH.

The UPLC/MS data demonstrated that the peak intensities of consecutive products (at m/z = 387, 359, 331, and 258) were first increased and then decreased gradually for α-MnO_2_ treatment at pH 3 and 4 (Fig. 4), indicating that the by-products formed under those conditions were unstable and finally degraded to a large extent. For comparison, the peak intensities of intermediates show a similar change for β-MnO_2_ and δ-MnO_2_ at pH 1 and pH 2, respectively (Fig. 4). However, the peak intensity of product (at m/z = 331) gradually increased during reaction time at pH 2 and pH 3 (Fig. 4), respectively, suggesting that RhB could not be degraded completely by β-MnO_2_ and δ-MnO_2_. These results reveal that the degradation mechanisms of RhB by the manganese oxides depend on the pH and crystal type.

## Conclusions

Three manganese oxides with different crystal type were prepared using reflux method to decolorize RhB in different pH solutions. The decolorization efficiencies of RhB for the three manganese oxides all increase with decrease solution pH. α-MnO_2_ presented in needles and showed the highest activity for RhB degradation at pH 2–6. δ-MnO_2_ and β-MnO_2_ had microsphere and rod-like form, respectively. These two manganese oxides showed activity to degrade RhB at pH 2–3 and only exhibited a very low adsorption rate and adsorption capacity at pH 4–6. The redox reaction between manganese oxides and RhB result in dissolution of manganese oxides and release of Mn(II). The degradation of RhB by manganese oxides consists of three main stages: (1) cleavage of the ethyl groups from RhB molecular to form Rh; (2) further destruction of –COOH and –CNH_2_ from Rh to form the small molecular substances; (3) mineralization of the small molecular substances into CO_2_, H_2_O, NO_3_^−^ and NH_4_^+^. These results indicate that manganese oxides especially α-MnO_2_ may have potential applications in degradation of dye pollutants.

## Methods

### Preparation of the manganese oxides

Cryptomelane (α-MnO_2_) was prepared through oxidation of Mn(II) by permanganate under refluxing condition [25]. Typically, 5.89 g of KMnO_4_ was dissolved in 100 mL of water and heated to boil, then a solution containing 8.8 g of MnSO_4_·H_2_O and 3 mL of concentrated HNO_3_ was added to the boiling solution. The mixture solution was refluxed for 24 h. The product was filtered, washed seven times with deionized water, and dried at 60°C for 24 h.

Pyrolusite (β-MnO_2_) was synthesized by refluxing process as reported in our previous work [12]. In a typical procedure, a mixture of 135 mL MnSO_4_ solution (1.75 mol/L) and 13.6 mL concentrated HNO_3_ (16 mol/L) was added quickly to 450 mL of boiled KMnO_4_ solution (0.04 mol/L). The resultant dark brown slurry was refluxed for 36 h, then filtered and washed with deionized water several times until the pH reached ~7.0. The final products were dried in an oven at 60°C for 24 h.

Birnessite (δ-MnO_2_) was prepared by refluxing treatment of KMnO_4_ and HCl mixture solution [26]. In a typical synthesis, 45 mL of 6 mol/L HCl was added to 400 mL of boiled KMnO_4_ solution (0.4 mol/L) at the rate of 0.7 L/min. The resultant dark brown slurry was refluxed for further 30 min. After being aged at 60°C for 12 h, the product was filtered, washed seven times with deionized water, and dried at 60°C for 24 h.

### Characterization of the prepared manganese oxides

X-ray powder diffraction (XRD) was carried out using a Bruker D8 ADVANCE X-ray diffractometer equipped with monochromated Cu Kα radiation (λ = 0.1541 nm) at a tube voltage of 40 kV and a tube current of 40 mA. Scanning electron microscopy (SEM) images were obtained with a Hitachi S-4800 emission scanning electron microscope. ASAP 2020 M+C instrument was used to measure the superficial area and micropore size distributions of the materials. Samples were degassed in a vacuum at 250°C for about 10 h to remove water and other physically adsorbed species. The N_2_ isothermal adsorption and desorption experiments were performed at relative pressures (P/P_0_) from 10^−6^ to 0.9916 and from 0.9916 to 0.047, respectively. The ζ-potential of manganese oxides were measured with an MALVERN ZEN 3600 electrophoretic light scattering spectrophotometer. The three manganese oxides dispersed into deionized water to form 0.5 g/L suspension solution and then treat it with ultrasonic for 60 min. Three suspension solutions were prefiltered through a 0.45 μm pore mill filter. The values of ζ-potential under different pH were measured.

The AOS of the three manganese oxide were measured by the oxalic acid-permanganate back-titration method. Briefly, 0.1 g of the samples were completely dissolved in 10 mL of 0.5 M H_2_C_2_O_4_ and 10 mL of 0.5 M H_2_SO_4_ to reduce all of the manganese to Mn^2+^. The extra C_2_O_4_^2−^ was determined by back-titration at 60°C with standardized 0.02 M KMnO_4_ solution. The AOS was calculated on the basis of both the titration result and the total amount of Mn determined by atomic absorption spectrophotometer (AAS) [27].

### Decolorization of RhB dye by the manganese oxides at various pH

The concentration of RhB solution was 10 mg/L, and the dosage of manganese oxides is 0.5 g/L. The solution pH was adjusted to set value by HCl and NaOH. The mixture was allowed to react in room temperature with continuous stirring. At given time intervals, an appropriate amount of suspension was taken out and quickly diluted the density to the point with distilled water. For optical absorption measurements, the diluted solution was immediately centrifuged at 12,000 rpm for 10 min to remove the manganese oxide particles. The changes of absorptions at 554 nm were applied to identify the concentrations of RhB, using a Shimadzu UV-2450 UV–vis spectrophotometer. The released of Mn(II) in the solutions were analyzed by AAS.

RhB and the intermediates generated in the degradation process were analyzed with ultra performance liquid chromatography (UPLC) triple quadrupole mass spectrometry (TQMS). Chromatographic separation was performed on a Waters Acquity UPLC system with a Crestpak C18S column that was placed at 40°C. The mobile phase was methanol/ultra pure water (1:1, v/v) at a flow rate of 0.5 mL/min. The sample injection volume was 10 μL. Mass spectrometry analysis was conducted on a Waters Aquity TQ Detector with electro spray ionization (ESI). The total organic carbon concentration in solution was analyzed using TOC analyzer (Shimadzu, TOC-vwp).

## Additional file

Additional file 1: The following additional data are availabe with the online version of this paper. **Figures S1–S3.** UV-visible absorbance spectra of RhB dye after different time intervals at various pH for α-MnO_2_, β-MnO_2_ and δ-MnO_2_. **Figure S4.** XRD patterns of the three manganese oxides before and after RhB bleaching. **Figure S5.** Ion chromatogram and Mass spectra of RhB intermediates. **Figure S6.** Degradation pathway of RhB by manganese oxides.
