# Supplementary material for: Decolorization of RhB dye by manganese oxides: effect of crystal type and solution pH
Source: Geochem Trans. 2015 Jul 25;16:10. doi: 10.1186/s12932-015-0024-2 (PMC4514904; doi:10.1186/s12932-015-0024-2)
Supplement: Additional file 1: — The following additional data are availabe with the online version of this paper. Figures S1–S3. UV-visible absorbance spectra of RhB dye after different time intervals at various pH for α-MnO2, β-MnO2 and δ-MnO2. Figure S4. XRD patterns of the three manganese oxides before and after RhB bleaching. Figure S5. Ion chromatogram and Mass spectra of RhB intermediates. Figure S6. Degradation pathway of RhB by manganese oxides. [file 12932_2015_24_MOESM1_ESM.docx]

**Additional files**

**Decolorization of RhB dye by manganese oxides****: Effect of crystal type and solution pH**

Hao-Jie Cui^1^, Hai-Zheng Huang ^2^, Baoling Yuan^3^, Ming-Lai Fu^1^*

^1^ Institute of Urban Environment, Chinese Academy of Sciences, Xiamen, 361021, China

^2^ College of Civil Engineering, Fuzhou University, Fuzhou, 350116, China

^3^ College of Civil Engineering, Huaqiao University, Xiamen, 361021, China

*Corresponding author: MLF

E-mail addresses: mlfu@iue.ac.cn

**Figure S1.** UV-visible absorbance spectra of RhB dye after different time intervals at various pH for α-MnO_2_.

**Figure S2.** UV-visible absorbance spectra of RhB dye after different time intervals at various pH for β-MnO_2_.

**Figure S3.** UV-visible absorbance spectra of RhB dye after different time intervals at various pH for δ-MnO_2_.










**Figure S4**. XRD patterns of the three manganese oxides before and after RhB bleaching.

**Figure S5.** Ion chromatogram and Mass spectra of RhB intermediates.


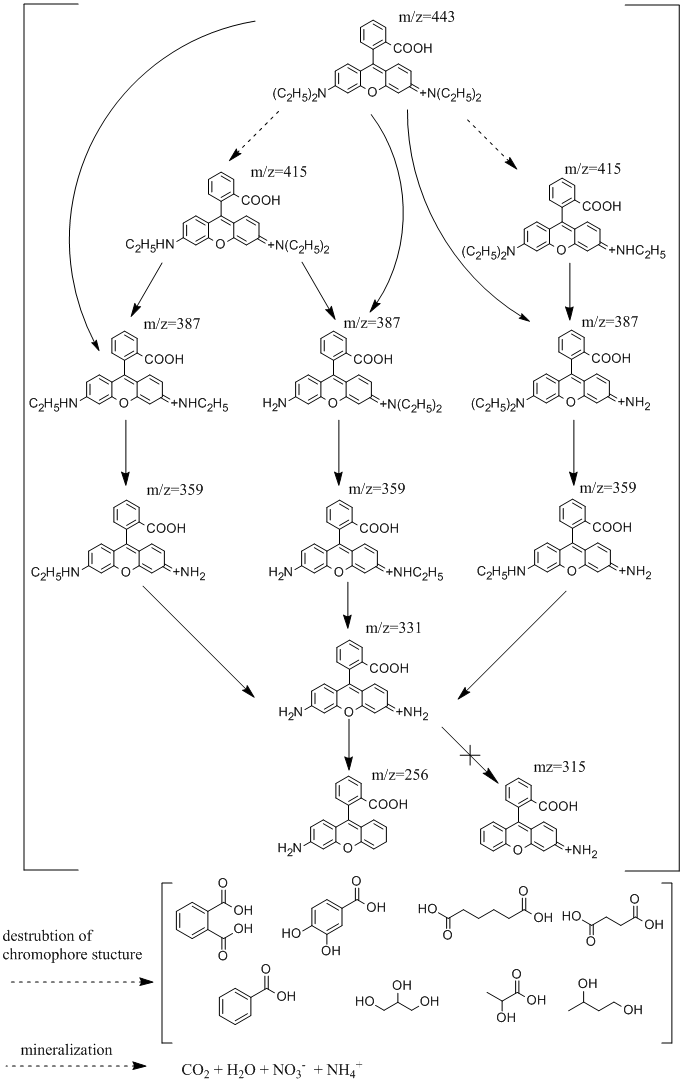


**Figure S6**. Degradation pathway of RhB by manganese oxides.
